# ‘It is like a tomato stall where someone can pick what he likes’: structure and practices of female sex work in Kampala, Uganda

**DOI:** 10.1186/1471-2458-13-741

**Published:** 2013-08-10

**Authors:** Martin Mbonye, Sarah Nakamanya, Winifred Nalukenge, Rachel King, Judith Vandepitte, Janet Seeley

**Affiliations:** 1 MRC/UVRI Uganda Research Unit on AIDS, P.O. Box 49, Entebbe, Uganda; 2 Department of Global Health, University of California, San Francisco, USA; 3 School of International Development, University of East Anglia, Norwich, UK; 4 London School of Hygiene and Tropical Medicine, London, UK

**Keywords:** Female sex work, Risk behaviour, Location, HIV, Uganda, Africa

## Abstract

**Background:**

Effective interventions among female sex workers require a thorough knowledge of the context of local sex industries. We explore the organisation of female sex work in a low socio-economic setting in Kampala, Uganda.

**Methods:**

We conducted a qualitative study with 101 participants selected from an epidemiological cohort of 1027 women at high risk of HIV in Kampala. Repeat in-depth life history and work practice interviews were conducted from March 2010 to June 2011. Context specific factors of female sex workers’ day-to-day lives were captured. Reported themes were identified and categorised inductively.

**Results:**

Of the 101 women, 58 were active self-identified sex workers operating in different locations within the area of study and nine had quit sex work. This paper focuses on these 67 women who gave information about their involvement in sex work. The majority had not gone beyond primary level of education and all had at least one child. Thirty one voluntarily disclosed that they were HIV-positive. Common sex work locations were streets/roadsides, bars and night clubs. Typically sex occurred in lodges near bars/night clubs, dark alleyways or car parking lots. Overall, women experienced sex work-related challenges at their work locations but these were more apparent in outdoor settings. These settings exposed women to violence, visibility to police, a stigmatising public as well as competition for clients, while bars provided some protection from these challenges. Older sex workers tended to prefer bars while the younger ones were mostly based on the streets. Alcohol consumption was a feature in all locations and women said it gave them courage and helped them to withstand the night chill. Condom use was determined by clients’ willingness, a woman’s level of sobriety or price offered.

**Conclusions:**

Sex work operates across a variety of locations in the study area in Kampala, with each presenting different strategies and challenges for those operating there. Risky practices are present in all locations although they are higher on the streets compared to other locations. Location specific interventions are required to address the complex challenges in sex work environments.

## Background

The Human Immunodeficiency Virus (HIV) remains a major public health threat in many Sub-Saharan African countries. Most studies in and outside this region show that the prevalence of HIV and sexually transmitted infections (STI) is much higher among sex workers compared to the general population, with some reporting rates of over 60% in some sex work populations [1-5]. In Uganda, by 2011, HIV prevalence among the general population in Kampala was 7.1% but female sex workers in the city were found to have a prevalence of 37% [5].

Research on sex workers has tended to focus on individual risk behaviours rather than on the underlying organizational factors that shape these risks [6]. The structure of the local sex work industries can give an indication of the specific vulnerabilities faced by the various people involved. While in some settings the legal status of sex work may afford support against some of the challenges faced by the women involved in the trade [7], in many countries in sub-Saharan Africa, sex work continues to be illegal [2,8]. The location of sex work may affect the degree of control sex workers have over their working environment, among other factors. Brothel based sex workers are reported to have more protection and better negotiating power compared to street based sex workers in many settings [2,9]. Although this is not always the case, as for example in Vietnam where it was reported that brothels were associated with higher risk than other locations [10]. Street based sex work is the most common and open form of sex work globally and the most dangerous in many areas, as women are exposed to violence by clients and the police, face social stigma and discrimination due to easy visibility and have to cope with a large volume of clients [7,8,11].

Women may have other jobs as well as providing sexual services, such as being waitresses in bars or petty traders [12,13]. In some of these jobs women may supplement their income by providing sexual services [14,15]. Women may engage in transactional sex from time to time when they need support, while others may do the work as their main occupation or one of their main activities [15]. This has made it hard to estimate the number of women engaged in sex work and even harder to provide any form of intervention for the many different categories of women [2,3].

Many sex workers use alcohol and illicit drugs as a coping mechanism against the emotional and physical challenges that they face while engaging with multiple sexual contacts. Alcohol consumption on a regular basis can increase their risk of violence as well as HIV infection because alcohol use is associated with inconsistent condom use [16].

The formal and informal organization of the sex work industry in sub-Saharan African settings hasnot been well described and this has hampered the use of such information for intervention possibilities, yet the prevalence of HIV continues to be worryingly high [5]. In this study we describe a setting in which women provide sex for money in a busy Kampala suburb, exploring the locations, types of women involved and the interaction with clients, to understand better, and potentially inform, interventions for this most at risk group of people.

### Study setting

The study was set in an area just outside the main Kampala city centre in two relatively poor suburbs that are dominated by one roomed houses, with some larger structures built along the roadside. There are many small businesses in this area with bars, retail shops and kiosks on the road sides selling general merchandise. More businesses operate in the evening with cooked food sellers, petty traders, including those selling alcohol, working on the roadside with some operating throughout the night. Night life in this part of Kampala is vibrant and alcohol consumption popular. Some of the retail shops which sell general merchandise during the day sell alcohol at night and compete with larger bars, night clubs and restaurants for customers. There are many guest houses and lodges which operate all day and night and provide space for paid sex.

## Methods

### Epidemiology cohort of women at high risk

The qualitative study on which we draw for this paper, was nested in a high risk cohort in Kampala [5]. The cohort was set up to study the epidemiology of HIV and other STIs among women involved in high risk sexual behaviours and to implement future HIV prevention trials. Women who were considered to be engaged in jobs that potentially exposed them to high risk of HIV and other sexually transmitted infections were recruited for follow up. The cohort started recruiting in 2008 and selected women who worked as bar maids, cooked food vendors, massage parlour workers, karaoke singers in bars, and restaurant waitresses. Consenting women were offered counselling and testing for HIV and other STIs, health education, free condoms, free access to primary health care and for those who were eligible for antiretroviral therapy, referred to providing centres. Those who had children under-five years of age received free medical care for these children at the cohort clinic. Women were recruited sequentially until a sample size of 1027 was arrived at over a period of a year.

### The qualitative sub-study

From March 2010 to June 2011, we conducted qualitative interviews with participants selected from the epidemiology cohort. All women from the cohort were eligible for the study. Every third woman presenting at the clinic was invited to join the study until 100 women had been selected. Given the diversity of the cohort this number allowed for a wide representation of the women, in order to capture different job categories, different age ranges and times at which the women were recruited as well as migration-pathways. Women who accepted to participate were given an appointment for the first of three in-depth interviews at which written informed consent was obtained. Three women refused to participate while three others who had earlier agreed to participate did not come for their interview and were replaced. However, one of the replaced women later presented for interview and expressed her willingness to participate, bringing the final sample to 101 women.

### Data collection

Data were collected through life history in-depth interviews using a topic guide covering themes around the women’s family backgrounds, relationship histories, and work history and current work practices. Data were captured during two to three interviews starting with an enrolment interview at which the women began to tell their life history (which was often augmented during the second interview). After this interview, a de-briefing exercise was organized by a senior social scientist with each interviewer for them to describe the interview experience and get feedback on gaps and areas for clarification. Any interesting issues for further probing were also noted which paved the way for the second interview. At the second interview, the life history was augmented, including sexual partnership histories where possible and work related data were collected. At this point participants were often more comfortable and disclosed information such as HIV status, current relationships and daily routines. It is at this interview that women reported whether they were engaged in sex work or not. For those who self-identified as sex workers, a further interview with a checklist that focused on the lives and livelihoods of women during sex work was conducted. This meant that only those women who had said that they were either currently involved in or had been involved in the past in sex work, could participate in this particular interview, and it is on these data this paper is based. Of the 101 women enrolled, 67 women were eligible for the third interview and of these 58 were actively engaged in sex work, while 9 women had since left sex work. We noted however that some women (N = 6) were borderline sex workers based on their narratives about how they occasionally engaged in transactional sex but they did not identify themselves as engaging in sex work.

All qualitative interviews were conducted in a private space close to the clinic, but not in that building. This was deliberately done to encourage free expression and emphasize the separation of the qualitative component from the cohort study. Four interviewers participated in the data collection and each recruited and interviewed a given set of participants throughout the study period, to strengthen rapport. No tape recorders were used on mutual agreement with the participants. The interviewers were all experienced social science team members with experience of qualitative interviews in different settings and were thoroughly trained in taking brief notes during interviews to expand into a full account immediately after each interview. The routine de-briefings were used to validate the data. Interviews lasted from between an hour to one and a half hours. Interviews were conducted in the local Luganda language which almost all participants were familiar with. Only one woman was interviewed in English since she was not very comfortable with Luganda.

### Data management and analysis

We used Nvivo 8 computer assisted qualitative software to manage the data. After the data had been typed in English, the four interviewers were assigned two scripts each to read and identify themes inductively. They then met and looked at each other’s themes and compared with the main analytical focus of the overall study to draw up a final coding framework which was used by all the team. The framework was tested again by comparing and discussing coded scripts. This process continued throughout the coding to promote consistency across the work of the team. Among the themes was material related to locations of sex work, challenges and opportunities within each location and what type of women preferred particular locations. Narratives about interactions with clients were also noted as another theme. These data were used as the basis for this paper. The quotations that appear in this paper were based on narratives that the research team felt illustrate the experience of the different women.

Ethical clearance was obtained from the Uganda Virus Research Institute’s Science and Ethics Committee and the Uganda National Council for Science and Technology. A transport refund of 5,000 shillings [about $2] was given and a bottle of soda provided during the interviews.

## Results

### Sample characteristics

The age range was 16 to 46 years. Forty four out of 67 women were from the majority Baganda ethnic group while three women were of Rwandese, Congolese and Sudanese origin respectively. The majority of women originated from places outside Kampala and had migrated to Kampala in search of work. However, they all told of coming from poor socio-economic backgrounds and struggling to find work. Most women had attended primary school with only a handful going beyond this level. As a result, many participants engaged (either historically or currently) in low paying unskilled jobs such as; working as housemaids, barmaids, food vending, restaurant waitresses and karaoke singing at night clubs.

The constant search for better prospects led them into sex work, usually on the advice from their peers and former workmates who talked of this more lucrative activity. Almost all women (N = 64) had at least one child with 34 women having three or more children to support, which was reported as a major reason for entry and persistence in sex work. Only one woman was officially married, the rest were single, separated or widowed. However, 37 women reported a regular partner although the term ‘regular’ was fluid and varied from woman to woman and in most cases these were short term money/support-motivated relationships which the women often hoped would persist. Thirty one out of 67 women disclosed that they were HIV- positive (this compares to the main cohort of 1027 women with an HIV-prevalence of 43% among those whose sole source of income was sex work and 33% among those who did sex work alongside other jobs). Forty two out of the 67 women reported sex work as the sole income generating activity whereas 16 reported supplementing it with other work. The other nine said that they had since left sex work giving reasons related to ill health, older age and influence of their regular male partners. However, returning to sex work was a real possibility given the history of unstable relationships reported in the life stories.

### Sex work locations

#### Streets/roadsides

Most women reported that they used the streets as a first step into sex work and only moved to other locations after gaining some experience. Spots near bars/entertainment places and night car parks offered a strategic location where women usually started waiting for clients as early as 6.00 pm. These spots were normally attached to facilities which also offered spaces for sex. On the streets, women stood in groups or individually depending on their preference. Typically the streets represented those places which were close to an entertainment place and which were likely to attract sizeable numbers of patrons on a regular basis. Women took strategic places where they could be visible to passing men and tried to market their services through gestures and calling out to potential clients who were passing by or who approached them for sexual services. Clients knew these sex work spots and a man would come to pick a woman of his choice and then negotiate directly with her. This is illustrated by one street based sex worker in the quotation below:

It is like a tomato stall where someone can pick what he likes, and everyone has a way she looks to attract a man because you cannot stand there and look like someone beaten and think that you can get a man. You have to put on a nice smile even if you are not happy, then a man can choose you quickly (29 year old street based sex worker).

Once a client identified a woman of his choice, the couple would negotiate the price and venue for sex. The price was mainly determined by agreed duration of the sexual act and whether a condom was to be used or not. Women usually charged 5,000 shillings [about $2] for quick sex which they normally referred to as *‘short’* time sex and this amounted to one ejaculation/round. This ‘short’ time sex on average lasted five and never exceeded 30 minutes. Unprotected sex cost more and the cost varied between sex workers depending on the woman’s level of need and work location. However, the client seemed to gain overall control of the woman upon paying for sexual services and street based women sometimes found it hard to sustain condom use after accepting money:

One cannot avoid non condom use because some men may take you after agreeing to use it but when they reach the room; they refuse to use condoms (27 year old street based sex worker).

Similarly, after negotiating sex, a client could sometimes offer a drink at a nearby bar and sometimes women drank too much and failed to use condoms:

It is only that there is no way you could insist on condom use; because you could be having fun and you get drunk and in that case, you cannot think about condoms or the man could even break the condom without you noticing (27 year old street based worker).

Another woman mentioned that alcohol use could cause violence and suggested that sometimes clients used alcohol for sinister motives:

There are times when you are drunk and you feel you want to fight or you have a high sexual desire. In such cases, you do not even think of condoms (19 year old street based sex worker).

She added that she tries to guard against HIV infection but she will probably get it accidentally.

Even without the offers from clients, women reported consuming alcohol while on the street which they carried in sachets and small bottles or bought from nearby bars to help them cope with the emotional side of the job and the night chill.

Much as clients were solicited on streets, sex usually took place in nearby lodges, dark alleyways or homes. Although women reported that they refrained from going to clients’ homes as a way of avoiding physical and sexual violence, financial demands sometimes made this impossible to maintain. They, however, rationalised this by charging much higher fees, as much as 10,000 shillings [roughly $4] and in order to ensure that this money was safe and to avoid possibilities of non-payment by the clients, women tended to demand cash before going with clients and they kept this money with a colleague for safe custody.

The women usually shared both good and bad experiences with each other about their clients and these served as a learning experience or an opportunity for women to focus on non-violent clients. Almost all sex work spots on streets/roadsides had a so called peer leader, usually an older and experienced sex worker or former sex worker who women referred to as ‘*senga’* (a Luganda term referring to a paternal aunt, who traditionally gives advice to her nieces on hygiene and sexual issues). In this case the term ‘*senga’* was used figuratively to suggest an older woman who had a leadership position and often oversaw the conduct of the women. This ‘*senga’* who was appointed by the sex workers tried to enforce some semblance of order amongst the group disciplining errant sex workers or settling disputes that came to her attention. To a large extent, in order to operate in a particular area, the sex workers were required to first report to this leader. They were then asked to pay a one-off fee of 30,000 to 50,000 shillings ($12 to $20) to be allowed to stand in a particular spot. This was an informal arrangement which was respected by the women as they entered into sex work. The peer leader would, in turn, introduce the new sex worker to others under her jurisdiction in that area. In rare cases, this came with some level of support in terms of finance in an emergency situation, advice or protection from police arrests, troublesome clients or settling disputes with other sex workers. Because sex work is illegal, women on the streets worried about police harassment. In some locations, women reported that they made weekly contributions of 500–1,000 Uganda shillings [less than $1] which helped keep the authorities at bay. Such women would then be protected as long as they remained within the given territory. Most women owned a mobile phone which they also used to keep contact with clients and other relevant persons for safety.

However, while staying in large groups had its advantages like providing safety against violence and possible exploitation by known clients, there were challenges of competition for clients and women had to keep employing innovative strategies to win clients. They might, for example, wear attractive and skimpy clothes, charge more attractive prices and use seductive language to attract men. This was often reported to have some challenges:

From one o’clock at night, I start moving to xxx [roadside location]. In that corridor, we are many women around that time chatting as we wait for clients. Clients do come because they know there are women to buy at that place. We compete for men as one [woman] could call out to a client but then another takes him. You then fight because that is a job, but you can sometimes get two at the same time and you give one to your friend, like that. [Laughs] Men come looking for prostitutes and when he is passing by, a woman calls out to that customer, ‘come and I offer you a cheap price’. (20 year old bar, street and phone based sex worker).

Some women said that they gained a monopoly over certain clients as some men expressed a preference for a particular sex worker. As an encounter with a client became more often, the relationship sometimes took on a more regular shape and condoms were often not used, especially if the clients had promised long term commitment. Women reported that although it was hard to conceal involvement in sex work while on streets, they saw some advantages in being on the street as they experienced high client volumes compared to indoor settings:

I prefer standing on the street as this is where I get more money. I could get like three to four clients who pay 5,000 shillings for a ‘short’ or even 10,000 for those who have money (19 year old street based sex worker).

### Bars/drinking places

Some sex workers reported getting their clients mainly in bars. The women described these bars as any place that sold alcohol or in some cases food. Typically the bars were reported to be of three types with the larger bars that targeted high income, corporate type customers, and the medium sized ones that targeted both the high and low income earners and others that were frequented by lower skilled workers. Bars with attached lodges or rooms for sex were common in areas where these women operated. More than half of the women who were over 30 years of age reported getting their clients exclusively in bars. Some also worked as bartenders while others went to bars individually or in groups, but each would sit at a table where she saw a man without the company of a woman, and then order a drink. The lone man would offer another drink and the conversation would start there. If the man proposed to have sex with her, that is when she would tell him to give her money and the negotiations would begin. A sex worker who also doubled as a waitress described how she got her clients:

When you are serving him food, he gives you his phone number and you also give him yours and he later calls you. Or when I go to drink in a bar, I sit at a table where men are seated and buy myself a drink. I stop at buying that one drink and the rest is bought for me. That is where I get men (39 year old bar based sex worker).

If a sex worker anticipated a more serious relationship with the man; she would not disclose her work but instead let him give whatever money he wishes. Negotiation for sex was between the woman and her client, with no third party involved except in a few cases of newly recruited sex workers who felt shy negotiating a price. However this was quite rare in bars where the women tended to have experience with negotiation.

Sitting inside bars helped conceal the intentions of women and they could disguise their involvement in sex work and also avoid arrest by the police. Often, these women said they feared walking on the streets and claimed to view street based sex work as lower status.

In bars, women charged higher prices with some reporting a minimum of 10,000 shillings ($4) for *‘short‘*time sex. As sex normally took place in lodges attached to the bars, a woman did not risk moving from her familiar territory and she could easily get help in case of violence from clients:

I prefer doing sex work in a bar because there are no problems; but on the streets, the police disturb women and arrest them which I have never experienced in a bar (30 year old bar based sex worker).

However, there were reports of physical and sexual violence when bar based sex workers agreed to go elsewhere and had sex with clients in spaces other than those attached to the bars.

### Lodges/guest houses

The lodges served as venues for sex after a client and sex worker agreed to a sexual encounter and either party could pay for the lodge depending on what they had agreed. Lodges were described as ranging from self-contained to single rooms with a well-made bed, a plastic wash basin and can of water normally placed under the bed. The rooms were lit with dim and coloured electric-light bulbs to create a welcome atmosphere and in the case of no power; a match box and a candle were provided. These lodges charged between 5,000 to 10,000 shillings [roughly $2-$4] depending on the quality. This fee often included a three condom packet but more condoms could be provided on request with an extra charge.

Typically, a woman would hire a room and move around searching for clients to bring to this room for paid sex. She would then make sure that she makes more money to cover the hiring fees and some extra to save. Lodge attendants were reported to sometimes have sex workers’ phone numbers and could link the sex worker to a client. This kind of arrangement was often organized at a woman’s request. In most cases, the money was paid directly to the sex worker although there were few reports of money being channelled through the attendants. Otherwise, there could be an exchange of phone contacts after the initial contact with a client and either party could call in case they wanted sex or money and the price was negotiated before the woman came to the agreed venue.

Lodge owners ensured the safety of the facility users by employing strong men who intervened in case of a violent client. Women reported leaving the lodge room door unlocked so they could easily get help if they felt threatened and raised an alarm.

### Dark corners/alleyways (*Covers*)

If a client did not have enough money to pay for a lodge or at the request of a woman who wanted to save lodging fees, sex could happen in a makeshift bathroom, against a wall, in a client’s car or a dark corner or alleyway. Women referred to these places as ‘*covers*’. The ‘*cover’* was at times a night security guard’s hut. With an intention of also earning extra money, the security guard placed card board on the floor which acted as a bed and he charged 1000 shillings (<$1) from those wanting to use his space for sex. In addition, security personnel guarding a market could let the couple have sex under the stalls at night. Other spaces that went for 1000 shillings (<$1) were in night car parks or car wash stands. A 32 year old street-based worker described her work place:

It is a car wash and the owner put an enclosure on one side and divided it up into compartments using papyrus mats. These compartments do not have doors on them and the papyrus mat is to prevent the other person from seeing you while you are having sex but does not prevent him/her from hearing what you are doing. Lanterns hanging on the wall provide the light. You pay 1,000 [less than half a $1] shillings at the entrance for every client that you take in there. The standard fee we normally charge [for sex] in this place is 3,000 shillings [slightly over $1] but if you are financially badly off, you could even charge 2,000 [<$1] and you pay 1,000 for the place and remain with 1,000 shillings. On some good days, you could get like six clients and retire with 10,000 [$4] or even 20,000 shillings [$8]; but these days you can even fail to raise 3,000 shillings a day because you could get just one client or even fail to get any. The place is also a night-car-park and the owner did not put a bar there because people park their cars and the drunkards could easily break the car windows […] but men know that it is a place for sex workers and do come in to buy sex. We even have bouncers who deal with troublesome men and such men know it and avoid this place. These bouncers are paid by the owner of the place.

She added that the compartments are so tiny and only accommodate a bed with a mattress made out of polythene sacks. The ‘bouncer’ or muscular man who was responsible for the security of the place provided a single condom in addition to collecting the space fees. This activity was practiced mainly by street-based sex workers. Women who used such places were more likely than women operating in other locations to report not using a condom.

### Private homes

In some very rare cases, sex could take place in a sex worker’s or client’s home, especially if it involved spending a night with the client. Women generally resisted going to clients’ homes to avoid the potential for violence or to maintain some power over the negotiations. Using private homes was mostly done with regular clients in whom women had developed trust. In this instance, either party could call and agree to meet. Women rarely reported going to first time clients’ homes and the few times this happened, it was after consulting with friends and getting some assurances that the client was not likely to be troublesome. There were some reports of violence in the form of physical/sexual assault, non-payment and non-condom use when sex happened in clients’ homes and this is why women avoided it with new clients. Sometimes they went against their own better judgement and risked going to clients’ homes or locations preferred by the client, if they really were desperate for money.

### Mobility across work locations

Many women were not restricted to a particular area for sex work but moved from one location to another based on event, season, time of the day and existing demand for clients. Some women reported moving from bars to streets and vice versa while others moved to different areas within and outside Kampala in search of clients. If a sex worker went from her usual area to work in a new one; she sat inside a bar like any other customer and solicited bar based clients to avoid competition-related violence from other sex workers who would not want her in their area. Within one night, a sex worker could operate in different locations around Kampala. The older sex workers who preferred operating in bars reported moving around different bars in case they failed to get clients in the first bar. Other sex workers, especially the younger ones not only operated in different suburbs of Kampala but moved from streets to bars and vice versa. Some women reported moving for one week/month to other towns or to the islands in Lake Victoria in search of better paying clients or following advice from experienced peers. We found that the women who reported moving to islands/landing sites were more likely to be HIV-infected. These women said that besides having lots of money at their disposal, fishermen did not like using condoms. So, there was always a greater possibility of having unprotected sex during these visits.

## Discussion

In this paper we have described the structure and organisation of commercial sex work in a Kampala suburb. Our particular focus has been on the different places the women work, and how those locations influence risk, including safety from violence as well as STI infection, and also the potential income of the women in each place. These findings are corroborated by research in other settings which highlight similar experiences for women [6,9,14,17].

Mobility was an important part of the lives of the women in our study. Not only did women vary their place of work, moving between the street, bars and lodges (depending on opportunity as well as their need for cash) but also by moving considerable distances to earn money. This travel may mean moving to a fishing site where the fish catch has been relatively good and the men have cash available or going to a workplace (construction site or oil palm plantation) on pay day. The risks involved in such mobility, because of high rates of STI infection, including HIV, at some of these sites highlighted in this study have been documented elsewhere [12,18-20].

Other factors are also important. The regular and sometimes excessive consumption of alcohol by both the women themselves during their work, as well as by their clients, was mentioned often in our interviews. The impact of alcohol consumption on sexual behaviour and risk taking, particularly the failure to use condoms, has been documented by a number of different commentators [21-25], as well as in our study population [26]. Because alcohol is both a means of coping with the provision of sexual services and an integral part of the entertainment industry which the sex workers also inhabit, interventions to reduce alcohol consumption are difficult to put in place in the absence of structural changes that may influence the availability and pricing of alcoholic drink.

The structural drivers of HIV infection, such as gender inequity, insecurity and poverty, pose a challenge for interventions with sex workers. These structural factors not only make negotiations over safer sex difficult for many women but also keep many women in sex work because of their need for cash to pay children’s school fees or simply to feed themselves and their children. Income generating opportunities may provide valuable alternatives for the day to day needs of a family, but when large expenses come, particularly when they are unexpected, selling sex may still be the most available way of raising cash. Structural changes often demand transformation in the wider society, but small steps to improving the conditions in which women work can be, and are, being made. There are well-documented examples of the success of approaches to address both the organisational and the legal status of sex workers in other places, most notably in Sonagachi, in India [27]. The informal organisations that some groups of women in our study population had put in place to support each other, as well as manage their relationships with the police and other authorities, may provide structures on which to build initiatives that support women’s rights as well as promote their safety and security.

Countering the violence and risk women face in their work, especially among street-based sex workers, may require confronting the legal issues around sex work [1,11]. However, such initiatives may not have the full impact desired. Some studies have shown that decriminalizing sex work may not result in improved conditions for all women engaged in sex work because of the many different situations in which women work, which are not easily regulated [7]. In addition, the mobility of the women in our study population, as we have shown in this paper, presents challenges for approaches that are tailored to address the needs of stationary populations.

Another challenge is the label ‘sex worker’. Van den Borne [28] describes in her work in Malawi the reasons why many women rejected the label ‘prostitute’; they were women in search of a man to provide support, they were not selling sex. Some, as in our study population, may have had multiple partners but these were often transactional relationships with regular partners, men who were not viewed as ‘clients’. Such women may face many of the same risks as sex workers in bars or lodges in terms of risks of infection, but also violence and abuse, but may not be reached by interventions which use labels or descriptions that they feel do not fit what they do [15,29]. It is very likely that some of the thirty four women in our study population who did not self-identify as sex workers fall into this category.

This study had some limitations which should be considered when interpreting the results. This was an urban population and may not give a picture of sex work in small towns or rural areas. Secondly, our sample was mainly from low socio-economic locations which may differ from high class sex work locations within the city centre.

## Conclusion

We have documented organizational patterns of sex workers in Kampala and noted the different challenges associated with the locations. The streets were places most commonly associated with violence and vulnerability among the sex workers. While the bars offered some sort of safety, the fluidity with which many women traversed the various locations means interventions tailored to the different settings are required. Given the high levels of alcohol consumption it is important to explore ways of heightening awareness of excessive alcohol consumption by working with bar owners, clients and sex workers themselves. Engaging the various key stake holders like the peer leaders (sengas), the police, bar owners, clients and local area leaders who understand the different settings in which sex work is organized would offer an opportunity for promoting safety for many of the women involved in this work.

## Competing interests

The authors declare that there are no competing interests.

## Authors’ contributions

JS and MM designed the study. WN and SN participated in data collection. SN and MM undertook data analysis, interpretation of the data and drafted the paper. RK, JV, JS reviewed and refined the manuscript. All authors read and approved the final manuscript.

## Pre-publication history

The pre-publication history for this paper can be accessed here:

http://www.biomedcentral.com/1471-2458/13/741/prepub
